# Proteasome-independent degradation of HIV-1 in naturally non-permissive human placental trophoblast cells

**DOI:** 10.1186/1742-4690-6-46

**Published:** 2009-05-15

**Authors:** Anna Laura Ross, Claude Cannou, Françoise Barré-Sinoussi, Elisabeth Menu

**Affiliations:** 1 Institut Pasteur, Unit of Regulation of Retroviral Infections, Department of Virology, 25 rue du Docteur Roux, Paris, France

## Abstract

**Background:**

The human placenta-derived cell line BeWo has been demonstrated to be restrictive to cell-free HIV-1 infection. BeWo cells are however permissive to infection by VSV-G pseudotyped HIV-1, which enters cells by a receptor-independent mechanism, and to infection by HIV-1 via a cell-to-cell route.

**Results:**

Here we analysed viral entry in wild type BeWo (CCR5^+^, CXCR4^+^) and BeWo-CD4^+ ^(CD4^+^, CCR5^+^, CXCR4^+^) cells. We report that HIV-1 internalisation is not restricted in either cell line. Levels of internalised p24 antigen between VSV-G HIV-1 pseudotypes and R5 or X4 virions were comparable. We next analysed the fate of internalised virions; X4 and R5 HIV-1 virions were less stable over time in BeWo cells than VSV-G HIV-1 pseudotypes. We then investigated the role of the proteasome in restricting cell-free HIV-1 infection in BeWo cells using proteasome inhibitors. We observed an increase in the levels of VSV-G pseudotyped HIV-1 infection in proteasome-inhibitor treated cells, but the infection by R5-Env or X4-Env pseudotyped virions remains restricted.

**Conclusion:**

Collectively these results suggest that cell-free HIV-1 infection encounters a surface block leading to a non-productive entry route, which either actively targets incoming virions for non-proteasomal degradation, and impedes their release into the cytoplasm, or causes the inactivation of mechanisms essential for viral replication.

## Background

The human immunodeficiency virus type 1 (HIV-1) must overcome and counteract a number of cellular factors to productively infect human cells. HIV-1 has evolved the ability to hijack several host molecules and mechanisms, thus using cellular factors as accomplices for viral infection. Although HIV-1 is able to most efficiently infect CD4 expressing T lymphocytes, other cell types can also be infected [1-3].

Conversely, many cell populations do not sustain HIV-1 replication either because they lack molecules essential for viral infection or they have restriction factors which can actively inhibit infection. A number of cellular proteins (notably APOBEC3G and TRIM5α) have been demonstrated to possess specific anti-viral properties and to be involved in restricting HIV-1 infection in non-permissive cells [4,5]. The cellular mechanism of proteasomal degradation has also been shown to play a role in restricting HIV-1 infection [6-8]. Several reports provide evidence supporting the role of proteasomal degradation, either in directly targeting incoming virions for degradation [6] or by modulating the cell cycle or cellular factors which are involved in viral infection [9]. The proteasome has been demonstrated to play a role in the TRIM5α-mediated restriction of HIV-1 in rhesus macaque monkey cells. Indeed, the treatment of rhesus cells with proteasome inhibitors resulted in an increase in the levels of HIV-1 reverse transcription. This did not, however, result in increased infectivity, leading to the hypothesis that there is a further restriction block in the viral cycle [6,10].

A number of reports suggested that human placental trophoblast cells may lack one or several essential host factors or be characterised by an active restriction factor which hinders HIV-1 infection in these cells. Human trophoblast cells form the outer barrier of the chorionic villi of the placenta, separating the maternal and fetal blood systems, and are therefore the first cells of the placenta to be exposed to cell-free or cell-associated virus in HIV^+ ^pregnant women [11]. The rate of early in uterotransmission of HIV-1 is relatively low indicating that the placenta acts as a physical barrier to the virus [12]. Transmission through the placenta could occur by direct infection, by transcytosis of the virus through placental cells [13], or via the passage of virions in physical breaks of the placental tissue [14]. Whereas it is clear that the placental tissue may be infected (either *in utero *or *in vitro*) by HIV-1, and that trophoblast cells are susceptible to infection by direct contact with infected PBMCs, the permissivity of trophoblast cells to cell-free virion infection remains a topic of much debate [15-18].

Various *in vitro *experimental models, such as the human choriocarcinoma BeWo cell line, have been used to study the mechanisms involved in trophoblast cell infection by HIV-1. Our studies and those from other groups have demonstrated that contact with HIV-1 infected cells leads to the infection of polarised monolayers of trophoblast cells [19,20]. However, *in vitro *models show that trophoblast cells are restricted for infection by cell-free HIV-1 [15]. Indeed, following exposure of trophoblast-derived BeWo cells to cell-free HIV-1 virions, no productive infection is detected. Even when trophoblast cell lines are exposed to cell-free virions in the presence of pro-inflammatory cytokines (such as TNF-α and IL-1, known to increase HIV-1 replication), a highly permissive reporter cell line was needed to detect the extremely low levels of viral production [17]. Following exposure to cell-free HIV-1 virions, the transformed BeWo trophoblast cell line does not contain any detectable integrated forms of HIV-1, indicating that the restriction to infection occurs at a viral step prior to integration [15]. BeWo cells are, however, capable of sustaining HIV-1 replication when transduced with the HIV-1 viral genome, or when infected with an HIV-1 pseudotyped virus bearing the G protein of the Vesicular Stomatitis Virus (VSV) [21,22], which does not require fusion for cell entry [23].

Primary trophoblast cells are known to express CCR5 and CXCR4, the two main co-receptors for HIV-1 entry [24,25]. Conversely, contradictory evidence exists concerning the expression of the main CD4 receptor on the surface of trophoblast cells, suggesting that CD4 may also be expressed, albeit at low levels; and, as for the two main co-receptors, CD4 expression may be regulated during pregnancy [25,26]. Published reports focusing on the utilisation of the CD4 receptor and the CCR5 and CXCR4 co-receptors for HIV-1 infection in trophoblast cells are contradictory: some evidence suggests that the virus makes use of the receptors for membrane fusion [27], while other reports suggest that HIV-1 entry in trophoblast cells occurs independently of the CD4 receptor [28] or via an, as yet undefined, endocytic pathway [27,29,30]. The BeWo cell line endogenously expresses the CCR5 and CXCR4 co-receptors, but does not express the CD4 molecule on the cell surface. For this reason, our group previously derived a CD4 expressing BeWo cell line to study HIV-1 infection. Despite the presence of the CD4 molecule as well as CCR5 and CXCR4, no cell-free HIV-1 infection was detected in this cell line [15]. However, we recently Recently our group has demonstrated that the CCR5 and CXCR4 co-receptors are implicated in cell-to-cell infection of trophoblast cells, as treatment with receptor antagonists drastically reduces HIV-1 infection [31]. The experimental evidence, therefore, indicates that trophoblast cell lines are restricted to HIV-1 cell-free infection and that this restriction likely affects the early steps of the viral life cycle.

The objective of the work described in this report is to gain insight into the mechanism of restriction of cell-free HIV-1 infection in the BeWo trophoblast cell line by investigating viral entry in both wild-type and CD4^+ ^cells as well as the potential role of proteasomal degradation.

## Results

### Cell-free HIV-1 infection is restricted in BeWo cells despite the presence of a functional CD4 receptor and CCR5 and CXCR4 co-receptors

**Figure 1:**
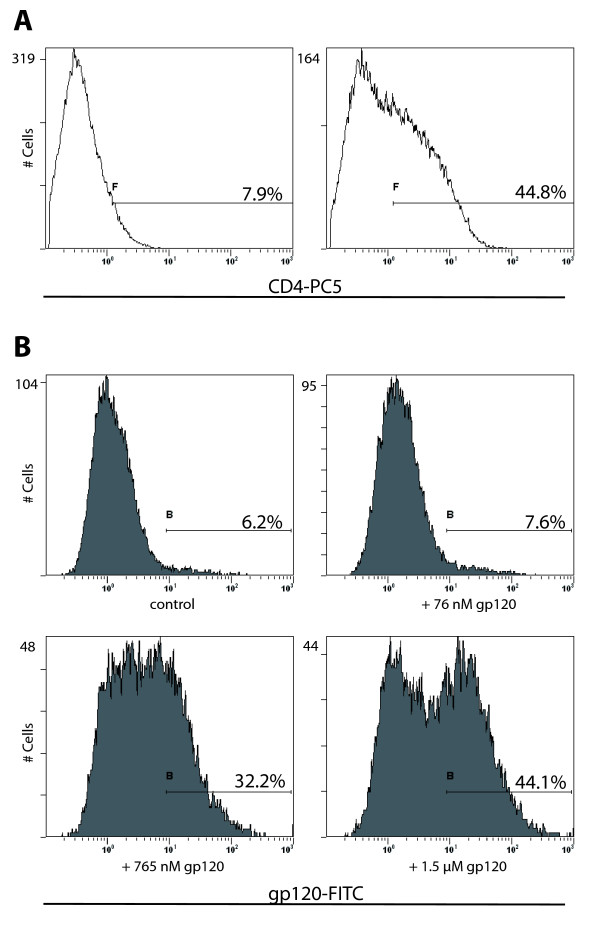
**The BeWo-CD4^+ ^cell line expresses CD4 capable of binding HIV-1 gp120 recombinant protein**. **A) **Expression of the CD4 surface molecule on the cell membrane of a selected BeWo cell line. The CD4 antibody used recognises the gp120 binding domain of CD4. The left panel shows wild type BeWo cells stained with anti-CD4 antibody; the right panel shows staining of the CD4^+ ^BeWo cell line. **B) **gp120-CD4 binding assays on the CD4^+ ^BeWo cell line. The CD4^+ ^cell line was incubated with increasing concentrations of recombinant gp120 protein and subsequently stained with an anti-gp120 antibody, followed by a FITC-conjugated secondary antibody; cells were analysed by flow cytometry. The gp120 recombinant protein is derived from a BaL HIV-1 strain.

As previously reported, cell-free HIV-1 infection remains restricted in the BeWo-CD4^+ ^cell line [15]. To ensure that the restriction in this stably transfected cell line is not due to a nonfunctional or incorrectly folded CD4 receptor, we analysed the binding capacity of the exogenously expressed CD4 receptor in the BeWo transfected cell line. To verify this functional aspect of the CD4 receptor in this transfected cell line, the cells were incubated with a CD4 antibody which specifically recognises the gp120 binding site of the receptor and the cells were then analysed by flow cytometry. The flow cytometry analyses indicated that approximately 40% of the BeWo-CD4^+ ^population expressed the CD4 surface molecule (Figure 1A). In order to ascertain the complete binding capacity of the exogenously expressed CD4 molecule, the BeWo-CD4^+ ^cells were used in a CD4-gp120 binding assay. Following incubation with recombinant gp120 (from HIV-1 BaL virus), the BeWo-CD4^+ ^cells showed a dose-dependent increase of fluorescently labeled cells (Figure 1B) compared to background levels in BeWo wild type cells (data not shown), indicating that the CD4 molecule in BeWo-CD4^+ ^cells is not only correctly expressed on the cell surface, but also capable of binding the HIV-1 gp120 envelope protein. However, despite a functional CD4 receptor and the endogenously expressed CCR5 and CXCR4 co-receptors, cell-free HIV-1 infection was still restricted in BeWo-CD4^+ ^cells (data not shown)[15].

### Viral internalisation in BeWo cells is envelope-independent

**Figure 2:**
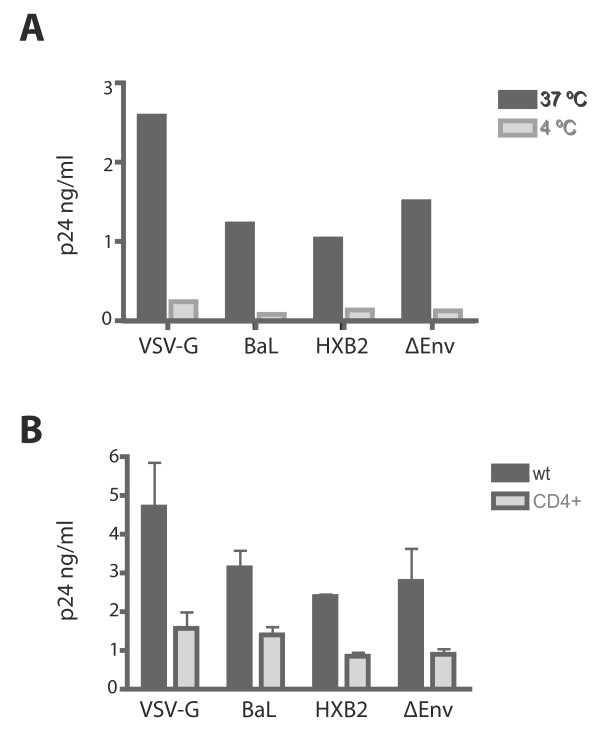
**HIV-1 pseudotypes, including envelope-deficient pseudotypes enter BeWo cells**. **A) **BeWo-CD4^+ ^cells were exposed to 20 ng/ml of p24 antigen of the HIV-1 pseudotypes at 37°C or 4°C by one hour spinoculation and one hour incubation at according temperature; the cells were washed, treated with pronase to eliminate residual virions on the cell surface, and the cell pellet was lysed in preparation for p24 antigen detection in lysates. Experiments were repeated at least three times. Graph shows a representative experiment. **B) **Wild type BeWo cells and CD4 expressing BeWo cells were exposed to HIV-1 pseudotypes. Viral entry was determined by p24 antigen quantification in cell lysates. The bars indicate the mean of at least three independent experiments; the error bars indicate SEM.

As cell-free infection by X4-Env and R5-Env pseudotyped HIV-1 is restricted in BeWo trophoblast cells despite the presence of functional CD4 receptor and co-receptors, we wished to verify that viral entry was not affected or diminished in these cells. The BeWo-CD4^+ ^were exposed to VSV-G pseudotyped HIV-1 as well as to X4 and R5 HIV-1 pseudotypes. An envelope deficient HIV-1 "virus" was also used as control. To determine the background level of virions attached to the cell surface, the experiments were performed in parallel at 37°C and at 4°C. As shown in figure 2A, the level of internalised HIV-1 (as determined by p24 antigen quantification) was significantly higher in the cells treated at 37°C than in the cells treated at 4°C. This result suggested that the small quantity of p24 antigen detected in the cell lysates exposed to the HIV-1 pseudotypes at 4°C was residual amounts of virions attached to the cell surface following pronase treatment and washes. The amount of intracellular p24 antigen in VSV-G infected BeWo-CD4^+ ^cells was slightly higher than the amount of intracellular p24 antigen following exposure to X4 or R5 viral pseudotypes. However, the amount of intracellular p24 antigen for X4 and R5 viruses was consistently substantially higher than the 4°C control (10–100 fold increase), indicating that the p24 antigen detected in the cell lysates was not simply bound to the cell surface of the BeWo-CD4^+ ^cells. Interestingly, even the cells exposed to an envelope-deficient HIV-1 pseudotype showed an internalisation of p24 antigen (figure 2A); this result suggested that the viral entry route most likely occurred via an endocytic pathway and not via membrane fusion. Having determined that viral entry occurs in the BeWo-CD4^+ ^cells, we performed the same experiments in the BeWo wild type cells. As shown in figure 2B, intracellular p24 antigen was also detected in wild type trophoblast cells (despite the absence of the CD4 receptor), and at even higher levels than those observed in the transfected BeWo-CD4^+ ^cell line.

### X4 and R5 HIV-1 viruses are less stable in trophoblast cells than infectious VSV-G HIV-1 pseudotypes

**Figure 3:**
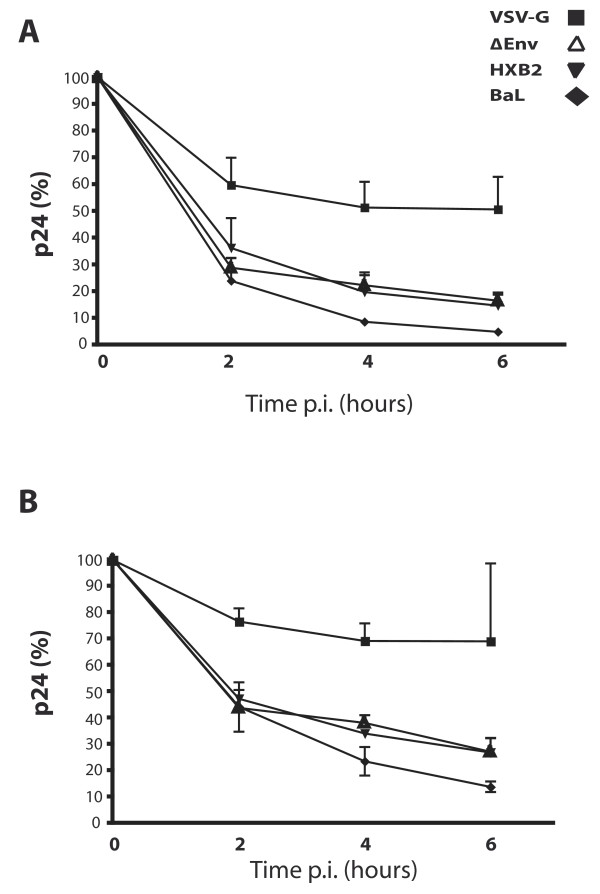
**VSV-G pseudotyped HIV-1 is more stable over time in BeWo wild type cells (A) and in BeWo-CD4^+ ^cells (B) compared to BaL, HXB2 and envelope-deficient HIV-1 virions**. Following viral exposure, the viral suspension was removed, and replaced with fresh medium; cell samples were collected at different time points. The amount of intracellular p24 antigen present in cells lysed immediately after viral exposure (time 0) was considered to be 100%, and all subsequent time points were normalised to time 0. The results are the mean of at least three independent experiments. Error bars indicate SEM.

Although HIV-1 particles are able to gain intracellular access to trophoblast cells, these cells are unable to sustain HIV-1 replication; we therefore analysed the fate of the virions once inside the BeWo cells. To follow the fate of intracellular p24 antigen, BeWo-CD4^+ ^cells were exposed to the different enveloped HIV-1 pseudotypes and infected as previously described. The intracellular levels of p24 antigen of the VSV-G pseudotype decrease, in the early time points, remained relatively stable in the BeWo cells (remaining at approximately 60% of total p24 antigen post-viral exposure) (Figure 3A). Conversely, the amount of intracellular p24 antigen for both the X4 (HXB2) and R5 (BaL) HIV-1 pseudotypes decreased more rapidly; and at 6 hours post-exposure, the detected level of remaining p24 antigen was less than 10% of the initial p24 antigen quantity (Figure 3A). The envelope-deficient viral genome (ΔEnv) was also tested in this assay, and its profile was found to be very similar to that of the enveloped HIV-1 pseudotypes. The fate of intracellular p24 antigen in BeWo wild type cells was also investigated; as previously determined, the absolute amount of p24 antigen was consistently higher in the wild type cells than in the BeWo-CD4^+ ^cells. However, the kinetics for the decrease of intracellular p24 antigen in BeWo wild type cells were very similar to those seen in the CD4 transfected cell line (Figure 3B).

### Intracellular p24 antigen levels of HIV-1 pseudotypes accumulated in proteasome-inhibitor treated BeWo cells

**Figure 4:**
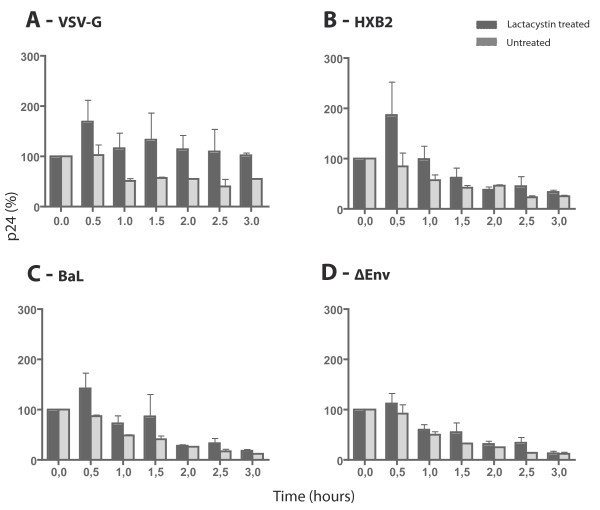
**The intracellular levels of p24 antigen of VSV-G, HXB2 and BaL enveloped HIV-1 pseudotypes accumulate in early time points in lactacystin-treated BeWo cells**. Intracellular p24 antigen levels for VSV-G **(A)**, HXB2 **(B)**, BaL **(C) **and envelope deficient **(D) **pseudotypes normalised against p24 antigen level at time point 0 (100%). BeWo cells were treated for 2 hours with 50 μM lactacystin prior to viral exposure, which greatly reduces proteasomal activity (as determined by the diminished release of the fluorogenic substrate Suc-LLVY-AMC; data not shown) without affecting cell viability (as determined by Wst-1 cell viability assays; data not shown). Bars indicate the mean of at least three independent experiments; error bars indicate SEM.

To determine whether the internalised virions are subject to degradation by the proteasome, the BeWo cells were treated with the proteasome inhibitor lactacystin. The lactacystin-treated BeWo cells were tested for optimal viability and proteasome inhibition levels; and consequently the cells were exposed to the HIV-1 pseudotypes as before, and the intracellular levels of p24 antigen were measured over-time (Figure 4). The data showed that the levels of p24 antigen in lactacystin-treated cells were higher in the early time points following exposure to both the HIV-1 enveloped pseudotypes as well as the VSV-G pseudotyped HIV-1. However, 3 hours post viral exposure, treatment with proteasome-inhibitors did not show any effect on the R5 and X4 HIV-1 pseudotypes, as their p24 antigen levels were similar in both treated and untreated cells. Conversely, the levels of intracellular p24 antigen for VSV-G HIV-1 exposed BeWo cells remained consistently higher over time (p = 0.0312) in lactacystin-treated cells compared to untreated cells.

### Proteasome inhibition in BeWo trophoblast cells did not overcome HIV-1 restriction

**Figure 5:**
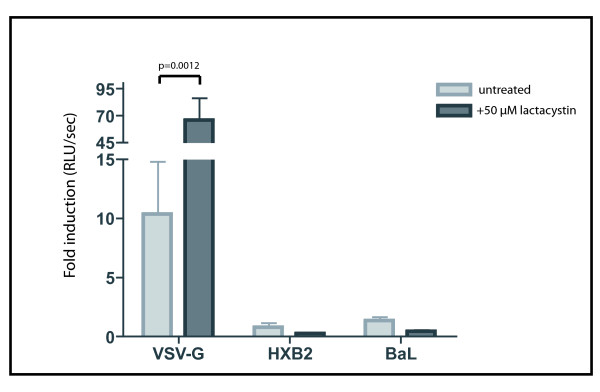
**The reduction of proteasomal degradation increases VSV-G pseudotyped HIV-1 replication, but not replication of HIV-1 enveloped pseudotypes**. VSV-G HIV-1 viral infection in lactacystin treated BeWo cells increases compared to untreated cells. The cells were exposed to the pseudotyped HIV-1 viruses (X4 and R5 pseudotypes, as well as the VSV-G pseudotype) and the level of infection in the cells was determined by the amount of luciferase activity detected in the cell lysates 96 hours post-infection. Results are expressed as the fold induction of luciferase activity as compared to uninfected cells. The results show mean of three independent experiments. Error bars indicate SEM. Statistical analyses were performed using Mann Whitney test.

To determine whether the accumulation of intracellular p24 antigen levels could constitute an escape mechanism for the restriction of viral replication in BeWo cells, we examined the effect of proteasome inhibition on viral replication. The level of luciferase activity detected in BeWo cells (both wild type and CD4^+^) infected with VSV-G HIV-1 was significantly higher (p = 0.002) in the cells which had been previously treated with the proteasome inhibitor (Figure 5). As expected, the untreated cells exposed to the X4 and R5 HIV-1 pseudotypes showed only background levels of luciferase activity; this baseline level of luciferase activity remained unaltered in the proteasome-inhibited cells (Figure 5). The experiments were repeated using two other proteasome inhibitors: epoxomicin and MG132. Similar to the results with lactacystin, VSV-G HIV-1 infection levels were increased, but no infection was detected for HXB2 and BaL enveloped HIV-1 pseudotypes (data not shown), suggesting that inhibition of proteasomal degradation is not sufficient to relieve HIV-1 restriction in BeWo cells.

## Discussion

The BeWo cell line is a standard *in vitro *model for the study of placental trophoblast cells. BeWo cells do not naturally express the CD4 molecule necessary for fusion entry of HIV-1 viral particles. Hence, to determine whether the restriction of cell-free HIV-1 infection is simply due to the lack of the main CD4 receptor, we previously transfected BeWo cells to obtain a CD4 expressing cell line which, however, also fails to sustain viral infection. Although the CD4 receptor was clearly expressed on the cell membrane, it was deemed important to verify that the receptor was capable of binding the gp120 protein of the HIV-1 envelope. We report that the CD4 molecule present on the surface of the BeWo trophoblast cells is capable of binding a recombinant HIV-1 BaL gp120 protein. Despite the gp120 binding capacity of CD4 and the functional expression of co-receptors, the trophoblast cells do not show any viral infection when exposed to the X4 and R5 HIV-1 pseudotypes. It has been suggested that the restriction of HIV infection in trophoblast cells is due to the lack of the appropriate receptors on the cell surface; indeed the levels of CD4 and the two co-receptors vary during pregnancy and several reports have confirmed the variable expression of CD4, CCR5, CXCR4 and other chemokine receptors [25,26]. However, while it is not possible to completely exclude a role of the CD4 receptor, the data reported here strengthen the hypothesis of a restriction in trophoblast cells which goes beyond the simple lack of receptor and co-receptor expression on the cell surface.

Given the controversial data on viral infection in trophoblast cells, it was important to determine whether viral entry is hindered in the BeWo cell line by comparing viral entry between the different HIV-1 pseudotypes and determine entry levels in wild type versus CD4^+ ^cells. Several studies have shown the significant role of an undefined endocytic pathway in virion internalisation in trophoblast cells [17,30]. However, despite virion internalisation, productive HIV-1 infection is restricted. Although virion internalisation by endocytosis pathways is generally associated with non-productive entry, there is evidence to show that HIV-1 entry via different endocytic processes can result in productive infection [32,33]. Our analyses show that HIV-1 virions gain intracellular access to both wild-type and CD4^+ ^BeWo cells. Interestingly our results also show that the levels of intracellular virus are similar, independent of the HIV-1 pseudotype used in the experiments (including an envelope deficient virus), and independent of whether the viral exposure leads to infection or not (VSV-G HIV-1 pseudotype compared to the HXB2 and BaL enveloped pseudotypes). The data indicate that the expression of the CD4 receptor is not necessary for viral entry, which likely occurs through an endocytic pathway, as suggested by others for different trophoblastic cell lines [27,29,30]. Given that all pseudotypes tested enter the BeWo cells at comparable levels, yet only the VSV-G bearing HIV-1 is capable of sustaining an infection, it is likely that the entry mechanisms or the very early post-entry events involved are different; and as a result, the intracellular compartmentalisation of the restricted pseudotypes versus VSV-G HIV-1 pseudotypes might be different. Intriguingly, the data described in this report demonstrate that the level of viral entry is higher in wild type BeWo cells (which do not naturally express the CD4 receptor) than in the CD4^+ ^transfected cell line. This observation could be explained by the fact that the CD4 receptor on BeWo cells has been demonstrated to bind the gp120 protein of HIV-1 (as shown in the protein binding assays), and therefore the exogenously expressed receptor may capture and sequester a portion of the HIV-1 virions, which are consequently no longer able to enter via a fusion-independent mechanism. Another potential explanation could be that the exogenous expression of the CD4 molecule on the cell surface could alter the natural characteristics of the cell membrane, reducing the levels of endocytosis, which in turn results in a decreased amount of internalised virus.

The data reported here indicate that all the HIV-1 pseudotypes tested enter the BeWo cells via an envelope-independent, non-fusion route at similar levels and kinetics, yet only the VSV-G bearing HIV-1 pseudotype can result in an infection. Following internalisation, all pseudotyped virus levels decrease to some extent, although the p24 antigen levels of VSV-G HIV-1 pseudotype are more stable than those of BaL, HXB2 or envelope-deficient HIV-1 pseudotypes. We hypothesized several non-mutually exclusive mechanisms which could explain the decrease of the p24 antigen levels of BaL and HXB2 enveloped HIV-1 pseudotypes. Once internalised by endocytosis, the restricted HIV-1 pseudotypes may be recycled and released from the cell by an exocytic pathway. Internalised virions may also be degraded by the proteasome or by lysosomes or they could be recognised by a restriction factor which impedes release into the cytoplasm preventing subsequent uncoating and reverse transcription.

The amount of p24 antigen detected in the culture supernatants following viral exposure increased slightly over-time (data not shown). This observation is consistent with the hypothesis of a portion of internalised virions being released by exocytosis. However, the low levels of p24 antigen detected in the supernatants more likely resulted from the detachment of membrane-bound particles. Thus the intracellular instability of the viral p24 antigen levels might not exclusively be due to release by exocytosis. The p24 antigen quantification experiments performed in the presence of the proteasome inhibitors demonstrated that a portion of the incoming virus was degraded by the proteasome. However the increased amount of internal p24 antigen in proteasome-inhibited cells for the R5 and X4 HIV-1 pseudotypes was only seen at the first time point measurements following infection. By 3 hours post viral exposure, the difference in p24 antigen levels of R5 and X4 HIV-1 viruses between proteasome-inhibitor treated cells and untreated cells was no longer detected. These data suggest that although the proteasome may contribute to the degradation of incoming virions immediately after viral exposure, the instability of internalised viral particles can also be due to other mechanism(s). On the contrary, when the proteasome-inhibited BeWo cells were exposed to the VSV-G HIV-1 pseudotype, the higher level of intracellular p24 antigen appears to be stable over-time. This increased level of internal p24 antigen of VSV-G HIV-1 seen in the lysates of proteasome-inhibited cells translates into an increase in the level of viral replication, as determined by the increase of luciferase activity following infection. Despite an initial increase of internal virus in BeWo cells exposed to HXB2 or BaL enveloped HIV-1 pseudotypes, the inhibition of proteasomal activity does not equate with an escape from the restriction of viral replication.

## Conclusion

In conclusion, although both VSV-G and HIV-1 pseudotyped virions all gained intracellular access to trophoblast cells with similar kinetics and comparable quantities, only VSV-G pseudotyped virions can productively infect these cells. The data presented here suggest that HIV-1 virions are engulfed by endocytosis, but in contrast with VSV-G enveloped HIV-1 virions, they are not released from the endocytic vesicles into the cytoplasm. A portion of internalised HIV-1 virions is subject to degradation by the proteasome, as demonstrated by the temporary increase of p24 antigen levels in proteasome-inhibited cell lysates. However, this transient increase is not sufficient to overcome HIV-1 restriction, and the virions are likely targeted for degradation by alternative mechanisms, such as the detrimental effect of the vacuolar pathway. Crucially, cell-to-cell HIV-1 infection is not restricted in trophoblast cells, bolstering the notion that the entry mechanism and early post-entry viral steps are critical in determining the restriction of cell-free HIV-1 virions. Direct contact with infected cells entrains separate entry mechanisms which allow HIV-1 virions to circumvent restriction in trophoblast cells. The notion that the transmission mode of HIV-1 may be key in determining restriction is highlighted by recent data on the ability of rhTRIM5α to restrict cell-free but not cell-associated HIV-1 transmission [34]. The results described in this report support the notion that trophoblast cells may recognise HIV-1 virions during the binding of the virus to the cell surface, internalise them in an endocytic, receptor-independent manner, and either actively target HIV-1 for degradation (which is principally proteasomal independent), or impede the release of the virions from the endosomes into the cytoplasm.

The elucidation of the mechanisms involved in HIV-1 infection and restriction of placental trophoblast cells remains of utmost importance to the understanding of the natural mechanisms of control of in uteroHIV-1 mother-to-child infection. This understanding holds potential implications for the identification of new therapeutic drug targets.

## Methods

### Cell lines

The human choriocarcinoma trophoblast cell line, BeWo [35] was obtained from the American Type Culture Collection (ATCC#CCL98, Rockville, Maryland, USA). A BeWo cell line stably transfected for the CD4 receptor was additionally used (BeWo-CD4^+^) [15]. BeWo cells were cultured in Dulbecco's Modified Eagle's Medium (DMEM, Gibco BRL), supplemented with 20% fetal calf serum (FCS). The titration of the viral pseudotype stocks was performed on HeLa P4P cells (CD4^+^, CCR5^+^, CXCR4^+^) cultured in DMEM supplemented with 10% FCS, containing 500 μg/ml G418 and 1 μg/ml puromycin. The 293T Human Embryonic Kidney cells (HEK) were used in transient transfections to obtain HIV-1 pseudotypes. The 293T HEK cells were cultured in DMEM, supplemented with 10% FCS.

### HIV-1 pseudotype infections

The luciferase expressing viral pseudotypes were based on the NL4-3 HIV-1 and VSV-G, BaL (R5) or HXB2 (X4) *env *plasmids [36-38]. The viral pseudotypes were generated by transfecting 293T cells with the corresponding cDNA plasmid (pNL4-3ΔEnvLuc+ plus the appropriate *env *cDNA) using the transfection reagent SuperFect (Qiagen) following the manufacturer's protocol. The resulting supernatants were collected 48–72 hours following transfection. The viral pseudotypes in the supernatants were quantified by p24 antigen ELISA (Zeptometrix) and titrated on HeLa P4P cells. The rate of infection of the viral pseudotypes was determined by luciferase expression using Luciferase Reagent (Promega) and a Glomax luminometer.

Viral infections were performed by treating 2 × 10^5 ^cells with 20 ng/ml p24 antigen of HIV-1 pseudotypes, followed by 1 hour spinoculation and 1 hour incubation at 37°C. Cells were subsequently incubated at 37°C for 72–96 hours, then washed in phosphate buffered saline (PBS) and lysed with 100 μl of Cell Lysis Buffer (Promega). 10 μl of cell lysates were used to determine levels of luciferase activity using the Luciferase Reagent (Promega) and reading was performed using Glomax Luminoter. Results are expressed as RLU/sec/100 μl lysate.

### CD4-gp120 binding assays

The gp120 binding assays were performed as previously described [39]. Both BeWo wild-type and BeWo-CD4^+ ^cells were incubated with different concentrations (76 nM, 765 nM, 1.5 μM) of recombinant HIV-1 BaL gp120 protein (NIH AIDS Research and Reference Reagent Program) for 4 hours at 4°C. Cells were washed twice in buffer (PBS, 1% FCS) and the cell pellet was resuspended in 50 μl of 2 μg/ml of anti-gp120 antibody (Aalto Bioreagents Ltd). After incubation for 1 hour at 4°C, the cells were washed and resuspended in 50 μl of anti-sheep IgG-FITC antibody (Sigma) and incubated for 1 hour at 4°C. Cells were washed and analysed by flow cytometry. All cell flow cytometry analysis was performed using Beckman Coulter DC500; results were analysed by CXP and FlowJo software.

### Viral entry assays

Cells were infected with viral pseudotypes as described above. BeWo cells were infected with R5 (BaL) or X4 (HXB2) viral pseudotypes or with envelope-deficient virus. Pseudotypes expressing the G protein of the Vesicular Stomatitis Virus (VSV), for which BeWo cells are permissive, were used as positive control. Following spinoculation and incubation, cells were washed twice with PBS and fresh medium added. Cells were then incubated at 37°C until cell lysis at the different time points. Cell lysates were prepared by washing cells in PBS, treatment with 1 mg/ml pronase for 3 minutes at room temperature to eliminate residual virus followed by two further washes in PBS. The cell pellet was resuspended in 100 μl of lysis buffer (Zeptometrix) and HIV-1 p24 antigen detection was performed using an ELISA assay (Zeptometrix).

### p24 antigen ELISA assays

The quantification of p24 antigen in cell lysates or supernatants was performed by p24 antigen ELISA (Zeptometrix), according to the manufacturer. The optical density of the samples was measured at 405 nm using Revel ELISA plate reader and software.

### Proteasome Inhibition

The proteasome inhibitor assays were performed using lactacystin (Sigma), epoxomicin (Sigma) and MG132 (Sigma). The effect of the proteasome inhibitors was determined by analysing the capability of the treated cells to release the fluorogenic substrate Suc-LLVY-AMC (Biomol International) compared to untreated cells. The cells were treated for 2 hours at 37°C with the appropriate concentration of proteasome inhibitor. After a 2 hour incubation period, the supernatant was removed, the cells were treated with lysis buffer and Suc-LLVY-AMC was added to the lysate. The fluorescence of the cells was measured using a Victor plate reader (Wallac). In parallel, the viability of the cells was tested by the Wst-1 assay (Boehringer Mannheim) to determine the toxicity of the proteasome inhibitors on the cells.

## Competing interests

The authors declare that they have no competing interests.

## Authors' contributions

ALR performed all experiments, participated in the design of the study and drafted the manuscript. CC provided technical assistance for experimental work. FBS participated in the conception and design of the project. EM participated in the design of the study, the conception of the project and helped draft the manuscript. All authors read and approved the final manuscript.

## References

[B1] Clapham PR, Weber JN, Whitby D, McIntosh K, Dalgleish AG, Maddon PJ, Deen KC, Sweet RW, Weiss RA (1989). Soluble CD4 blocks the infectivity of diverse strains of HIV and SIV for T cells and monocytes but not for brain and muscle cells. Nature.

[B2] Yahi N, Baghdiguian S, Moreau H, Fantini J (1992). Galactosyl ceramide (or a closely related molecule) is the receptor for human immunodeficiency virus type 1 on human colon epithelial HT29 cells. Journal of Virology.

[B3] Schneider-Schaulies J, Schneider-Schaulies S, Brinkmann R, Tas P, Halbrügge M, Walter U, Holmes HC, Ter Meulen V (1992). HIV-1 gp120 receptor on CD4-negative brain cells activates a tyrosine kinase. Virology.

[B4] Sheehy AM, Gaddis NC, Choi JD, Malim MH (2002). Isolation of a human gene that inhibits HIV-1 infection and is suppressed by the viral Vif protein. Nature.

[B5] Stremlau M, Owens C, Perron M, Kiessling M, Autissier P, Sodroski J (2004). The cytoplasmic body component TRIM5α restricts HIV-1 infection in Old World monkeys. Nature.

[B6] Anderson JL, Campbell EM, Wu X, Vandegraaff N, Engelman A, Hope TJ (2006). Proteasome inhibition reveals that a functional preintegration complex intermediate can be generated during restriction by diverse TRIM5 proteins. Journal of Virology.

[B7] Chatterji U, Bobardt MD, Gaskill P, Sheeter D, Fox H, Gallay PA (2006). Trim5alpha accelerates degradation of cytosolic capsid associated with productive HIV-1 entry. J Biol Chem.

[B8] Sakuma R, Noser JA, Ohmine S, Ikeda Y (2007). Inhibition of HIV-1 replication by simian restriction factors, TRIM5 alpha and APOBEC3G.

[B9] Dueck M, Guatelli J (2007). Evidence against a direct antiviral activity of the proteasome during the early steps of HIV-1 replication. Virology.

[B10] Campbell EM, Perez O, Anderson JL, Hope TJ (2008). Visualization of a proteasome-independent intermediate during restriction of HIV-1 by rhesus TRIM5alpha. J Cell Biol.

[B11] Burton GJ, Watson AL (1997). The Structure of the Human Placenta: Implications for Initiating and Defending Against Virus Infections. Rev Med Virol.

[B12] Chouquet C, Burgard M, Richardson S, Rouzioux C, Costagliola D (1997). Timing of mother-to-child HIV-1 transmission and diagnosis of infection based on polymerase chain reaction in the neonatal period by a non-parametric method. AIDS.

[B13] Lagaye S, Derrien M, Menu E, Coïto C, Tresoldi E, Mauclère P, Scarlatti G, Chaouat G, Barré-Sinoussi F, Bomsel M (2001). HIV-1 ENftSoIUTo: Cell-to-cell contact results in a selective translocation of maternal human immunodeficiency virus type 1 quasispecies across a trophoblastic barrier by both transcytosis and infection. Journal of Virology.

[B14] Burton GJ, O'Shea S, Rostron T, Mullen JE, Aiyer S, Skepper JN, Smith R, Banatvala J (1996). Physical breaks in the placental trophoblastic surface: significance in vertical transmission of HIV. AIDS.

[B15] Dolcini G, Derrien M, Chaouat G, Barré-Sinoussi F, Menu E (2003). Cell-free HIV type 1 infection is restricted in the human trophoblast choriocarcinoma BeWo cell line, even with expression of CD4, CXCR4 and CCR5. AIDS Res Hum Retroviruses.

[B16] Muñoz LD, Serramía MJ, Fresno M, Muñoz-Fernández MA (2007). Progesterone inhibits HIV-1 replication in human trophoblast cells through inhibition of autocrine tumor necrosis factor secretion. J Infect Dis.

[B17] Vidricaire G, Tardif MR, Tremblay MJ (2003). The low viral production in trophoblastic cells is due to a high endocytic internalization of the human immunodeficiency virus type 1 and can be overcome by the pro-inflammatory cytokines tumor necrosis factor-alpha and interleukin-1. J Biol Chem.

[B18] Bourinbaiar AS, Nagorny R (1993). Human immunodeficiency virus type 1 infection of choriocarcinoma-derived trophoblasts. Acta Virol.

[B19] Derrien M, Faye A, Dolcini G, Chaouat G, Barre-Sinoussi F, Menu E (2005). Impact of the placental ctokine-chemokine balance on regulation of cell-cell contact-induced human immunodeficiency virus type 1 translocation across a trophoblastic barrier in vitro. Journal Of Virology.

[B20] Vidricaire G, Tremblay MJ (2005). Rab5 and Rab7, but not ARF6, govern the early events of HIV-1 infection in polarized human placental cells. J Immunol.

[B21] Kilani RT, Chang LJ, Garcia-Lloret MI, Hemmings D, Winkler-Lowen B, Guilbert LJ (1997). Placental trophoblasts resist infection by multiple human immunodeficiency virus (HIV) type 1 variants even with cytomegalovirus coinfection but support HIV replication after provirus transfection. Journal of Virology.

[B22] Zachar V, Spire B, Hirsch I, Chermann JC, Ebbesen P (1991). Human transformed trophoblast-derived cells lacking CD4 receptor exhibit restricted permissiveness for human immunodeficiency virus type 1. Journal of Virology.

[B23] Roberts PC, Kipperman T, Compans RW (1999). Vesicular stomatitis virus G protein acquires pH-independent fusion activity during transport in a polarized endometrial cell line. Journal of Virology.

[B24] Kumar A, Kumar S, Dinda AK, Luthra K (2004). Differential expression of CXCR4 receptor in early and term human placenta. Placenta.

[B25] Mognetti B, Moussa M, Croitoru J, Menu E, Dormont D, Roques P, Chaouat G (2000). HIV-1 co-receptor expression on trophoblastic cells from early placentas and permissivity to infection by several HIV-1 primary isolates. Clinical And Experimental Immunology.

[B26] David FJ, Autran B, Tran HC, Menu E, Raphael M, Debre P, Hsi BL, Wegman TG, Barre-Sinoussi F, Chaouat G (1992). Human trophoblast cells express CD4 and are permissive for productive infection with HIV-1. Clin Exp Immunol.

[B27] David FJ, Tran HC, Serpente N, Autran B, Vaquero C, Djian V, Menu E, Barré-Sinoussi F, Chaouat G (1995). HIV infection of choriocarcinoma cell lines derived from human placenta: the role of membrane CD4 and Fc-Rs into HIV entry. Virology.

[B28] Zachar V, Nørskov-Lauritsen N, Juhl C, Spire B, Chermann JC, Ebbesen P (1991). Susceptibility of cultured human trophoblasts to infection with human immunodeficiency virus type 1. J Gen Virol.

[B29] Vidricaire G, Imbeault M, Tremblay MJ (2004). Endocytic host cell machinery plays a dominant role in intracellular trafficking of incoming human immunodeficiency virus type 1 in human placental trophoblasts. Journal of Virology.

[B30] Vidricaire G, Tremblay MJ (2007). A clathrin, caveolae, and dynamin-independent endocytic pathway requiring free membrane cholesterol drives HIV-1 internalization and infection in polarized trophoblastic cells. Journal of Molecular Biology.

[B31] Ayouba A, Cannou C, Nugeyre MT, Barre-Sinoussi F, Menu E (2008). Distinct efficacy of HIV-1 entry inhibitors to prevent cell-to-cell transfer of R5 and X4 viruses across a human placental trophoblast barrier in a reconstitution model in vitro. Retrovirology.

[B32] Daecke J, Fackler OT, Dittmar MT, Krausslich HG (2005). Involvement of clathrin-mediated endocytosis in human immunodeficiency virus type 1 entry. J Virol.

[B33] Maréchal V, Prevost MC, Petit C, Perret E, Heard JM, Schwartz O (2001). Human immunodeficiency virus type 1 entry into macrophages mediated by macropinocytosis. Journal of Virology.

[B34] Richardson MW, Carroll RG, Stremlau M, Korokhov N, Humeau LM, Silvestri G, Sodroski J, Riley JL (2008). Mode of Transmission Affects the Sensitivity of HIV-1 to Restriction by Rhesus TRIM5{alpha}. J Virol.

[B35] Pattillo RA, Gey GO (1968). The establishment of a cell line of human hormone-synthesizing trophoblastic cells in vitro. Cancer Res.

[B36] Akkina RK, Walton RM, Chen ML, Li QX, Planelles V, Chen IS (1996). High-efficiency gene transfer into CD34+ cells with a human immunodeficiency virus type 1-based retroviral vector pseudotyped with vesicular stomatitis virus envelope glycoprotein G. Journal of Virology.

[B37] Connor RI, Chen BK, Choe S, Landau NR (1995). Vpr is required for efficient replication of human immunodeficiency virus type-1 in mononuclear phagocytes. Virology.

[B38] Landau NR, Page KA, Littman DR (1991). Pseudotyping with human T-cell leukemia virus type I broadens the human immunodeficiency virus host range. Journal of Virology.

[B39] Mondor I, Ugolini S, Sattentau QJ (1998). Human immunodeficiency virus type 1 attachment to HeLa CD4 cells is CD4 independent and gp120 dependent and requires cell surface heparans. J Virol.

